# tACS Phase Locking of Frontal Midline Theta Oscillations Disrupts Working Memory Performance

**DOI:** 10.3389/fncel.2016.00120

**Published:** 2016-05-06

**Authors:** Bankim S. Chander, Matthias Witkowski, Christoph Braun, Stephen E. Robinson, Jan Born, Leonardo G. Cohen, Niels Birbaumer, Surjo R. Soekadar

**Affiliations:** ^1^Applied Neurotechnology Lab, Department of Psychiatry and Psychotherapy, University Hospital of TübingenTübingen, Germany; ^2^MEG Center, University Hospital of TübingenTübingen, Germany; ^3^CIMeC, Center for Mind/Brain Sciences, University of TrentoTrento, Italy; ^4^National Institute of Mental Health (NIMH), MEG Core FacilityBethesda, MD, USA; ^5^Institute of Medical Psychology and Behavioral Neurobiology, University of TübingenTübingen, Germany; ^6^National Institute of Neurological Disorders and Stroke (NINDS)Bethesda, MD, USA

**Keywords:** frontal midline theta (FMT), entrainment, transcranial alternating current stimulation (tACS), magnetoencephalography (MEG), working memory performance

## Abstract

**Background:** Frontal midline theta (FMT) oscillations (4–8 Hz) are strongly related to cognitive and executive control during mental tasks such as memory processing, arithmetic problem solving or sustained attention. While maintenance of temporal order information during a working memory (WM) task was recently linked to FMT phase, a positive correlation between FMT power, WM demand and WM performance was shown. However, the relationship between these measures is not well understood, and it is unknown whether purposeful FMT phase manipulation during a WM task impacts FMT power and WM performance. Here we present evidence that FMT phase manipulation mediated by transcranial alternating current stimulation (tACS) can block WM demand-related FMT power increase (FMTΔpower) and disrupt normal WM performance.

**Methods:** Twenty healthy volunteers were assigned to one of two groups (group A, group B) and performed a 2-back task across a baseline block (block 1) and an intervention block (block 2) while 275-sensor magnetoencephalography (MEG) was recorded. After no stimulation was applied during block 1, participants in group A received tACS oscillating at their individual FMT frequency over the prefrontal cortex (PFC) while group B received sham stimulation during block 2. After assessing and mapping phase locking values (PLV) between the tACS signal and brain oscillatory activity across the whole brain, FMT power and WM performance were assessed and compared between blocks and groups.

**Results:** During block 2 of group A but not B, FMT oscillations showed increased PLV across task-related cortical areas underneath the frontal tACS electrode. While WM task-related FMTΔpower and WM performance were comparable across groups in block 1, tACS resulted in lower FMTΔpower and WM performance compared to sham stimulation in block 2.

**Conclusion:** tACS-related manipulation of FMT phase can disrupt WM performance and influence WM task-related FMTΔpower. This finding may have important implications for the treatment of brain disorders such as depression and attention deficit disorder associated with abnormal regulation of FMT activity or disorders characterized by dysfunctional coupling of brain activity, e.g., epilepsy, Alzheimer’s or Parkinson’s disease (AD/PD).

## Introduction

Brain oscillations reflect rhythmic fluctuations in neuronal excitability modulating long-range communication between cortical and subcortical areas important for cognition and adaptive behavior in humans (Buzsáki and Draguhn, 2004). Cognitive functions such as memory processing (Düzel et al., 2010; Hanslmayr and Staudigl, 2014), arithmetic problem solving (De Smedt et al., 2009) and sustained attention (for review, see Clayton et al., 2015) required for many working memory (WM) tasks were linked to theta (4–8 Hz) and gamma (>30 Hz) frequency oscillations (Buzsáki, 2006) in a subcortical-cortical network that includes the hippocampus and fronto-medial brain regions. Fronto-medial theta (FMT) oscillations (Ishihara and Yoshi, 1972) were particularly attributed to the prefrontal and anterior cingulate cortex (PFC, ACC), the latter being a highly interconnected brain area shown to be involved in various cognitive and executive functions (Beckmann et al., 2009; Vogt, 2009; Cavanagh et al., 2012). Recently, a positive correlation between FMT power and WM demand was shown (Gevins et al., 1997; Jensen and Tesche, 2002; Mitchell and Cusack, 2008; Brookes et al., 2011), but the underlying mechanisms and functional significance of such FMT power increase (FMTΔpower) and its relatedness to FMT phase are still widely unknown (Hsieh and Ranganath, 2014).

While it was shown that the amplitude of brain oscillations that can be assessed as signal (SE) power at a certain frequency, e.g., in the theta band, reflects the number of simultaneously activated neurons, the phase of these brain oscillations provides information about the timing of the underlying neuronal activity (Sauseng and Klimesch, 2008). Thus, FMT power and FMT phase provide complementary information. Recently it was suggested that maintenance of temporal order information, as required e.g., in an *n*-back task, influences the length of theta cycles resulting in a shift of FMT towards lower frequencies along with increasing WM demand (Axmacher et al., 2010). Regulation of such cycles was attributed to recurrent anatomical pathways between the hippocampus and PFC (Blatow et al., 2003) with PFC activity driven by hippocampal theta phase rather than vice-versa (Anderson et al., 2010; Gordon, 2011). Based on the complementary learning systems model (Norman et al., 2005), increased FMT power during execution of an attention-demanding task with high cognitive load, e.g., during execution of an *n*-back task, on the other hand, was mainly attributed to increased inhibition of competing cortical representations related to previous WM trials (Norman et al., 2007). Correspondingly, absence of FMTΔpower during higher WM demand was reported to be associated with reduced performance (Donkers et al., 2011). However, it was unclear how FMT phase and FMT power relate to each other, and whether purposeful modulation of FMT phase can be used to target regulation of FMT power and influence cognitive control. Such knowledge would be important to gain deeper understanding of many brain disorders in which regulation of FMT oscillations is disturbed, e.g., depression (Olbrich and Arns, 2013), Alzheimers disease (AD) (Moretti et al., 2009; Laske et al., 2015) or attention deficit and hyperactivity disorder (ADHD; Missonnier et al., 2013).

Here, we investigated whether external manipulation of FMT phase during a WM task influences modulation of FMT power and WM performance. We hypothesized that manipulation of the physiological FMT phase regulation in frontal cortical regions interferes with maintenance of cortical representations related to previous WM trials and thus, results in lack of FMTΔpower associated with increase WM demand as well as disruption of normal WM performance.

To test this hypothesis, we used amplitude-modulated transcranial alternating current stimulation (tACS; Witkowski et al., 2015), a non-invasive brain stimulation (NIBS) method that can be used to modulate neural oscillations in a frequency-specific manner. In tACS, a weak (usually ≤2 mA) alternating electric current between two or more electrodes attached to the scalp is delivered. *In vitro* (Fröhlich and McCormick, 2010) and *in vivo* (Ali et al., 2013) studies have recently demonstrated that application of alternating currents can result in phase-alignment between neuronal spiking activity and the applied electrical stimulation signal (for review, see Veniero et al., 2015; Woods et al., 2016). Such tACS-based entrainment of brain oscillatory activity was shown to be accompanied by periodic fluctuations in behavioral performance providing further evidence for a causal relationship between brain oscillations and behavior (Thut et al., 2012).

Recently, we have shown that transcranial electric currents can be applied during assessment of whole-head magnetoencephalography (MEG; Soekadar et al., 2013; Garcia-Cossio et al., 2015) and that tACS-entrained cortical oscillation can be mapped at millimeter precision using amplitude-modulated stimulation signals (Witkowski et al., 2015). We reasoned that tACS oscillating at the individual FMT frequency delivered over frontal cortical regions during execution of an n-back task would lead to FMT phase-locked activity blocking hippocampus-driven phase regulation and thus, inhibition of competing cortical representations required for successful execution of a demanding WM task.

The possibility to record whole-head neuromagnetic activity during transcranial electric stimulation allowed us to verify success of the intended external manipulation of brain oscillations while, at the same time, allowing us to assess and map immediate effects of such manipulation on brain oscillatory activity throughout various brain areas and evaluate its impact on cognition and adaptive behavior.

## Materials and Methods

### Study Participants

Twenty healthy volunteers (4 females, all right handed, mean Age = 27.4 ± 3.25 years) without a history of neurological or psychiatric disorders were invited to the MEG center of the University of Tübingen, Germany. The study was approved by the University’s Ethics Committee (No.401/2012BO1) and written informed consent was obtained from all participants prior to their inclusion in the study.

### Experimental Design

**Figure 1:**
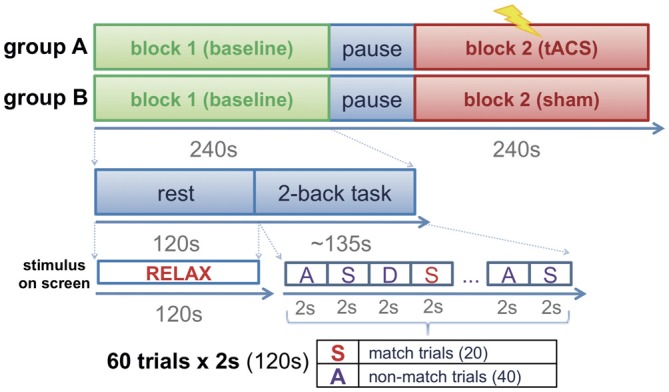
**Experimental design.** Participants were assigned to one of two groups (group A: transcranial alternating current stimulation (tACS); group B: sham stimulation). The experimental session consisted of two blocks (block 1: baseline block; block 2: interventional block). While group A received tACS during block 2, group B performed the task in absence of stimulation (sham stimulation).

Participants were assigned to one of two groups (group A, group B) in a pseudo-randomized order following a counter balanced factorial design. Before the experimental session, all participants were invited to a familiarization session in which they were accustomed to the setup and task. FMT activity was recorded to calculate the frequency of the individual maximum theta peak in each participant. The experimental session consisted of two blocks, a baseline block (block 1) and an intervention block (block 2). Each block had a duration of approximately 4 min and consisted of 2 min of rest (task-free state) during which participants had their eyes open fixating a cross visualized on a screen in front of them, and approximately 2 min of task during which participants performed a 2-back task across two runs (Figure 1) separated by a short brake of 10–15 s. Each run consisted of 30 trials (2 s each) resulting in a total of 60 trials per block. During the task, letters were presented on a screen in front of the participants at an inter-stimulus interval of 2 s. For stimulus presentation, Psychotoolbox was used. The participants were instructed to indicate whether the displayed letter matched with the 2nd letter previously shown by pressing a button with their left index finger, or to indicate that the letter did not match by pressing a button with their right index finger. A total of 30 letters were shown in each of the two runs. Only 10 of these letters in each run randomly matched with the 2nd previous letter (match trials) resulting in 20 possible correct answers (match trials) per block (Figure 1). While group A received tACS amplitude-modulated at the individual theta peak frequency during the whole duration of block 2, sham stimulation was delivered during block 2 of group B.

### Magnetic Resonance Imaging (MRI)

Before the first MEG session, participants were invited for a cranial magnetic resonance (MR) imaging exam using a 3-tesla whole body scanner with a 12-channel head coil (Magnetom Trio^®^, Siemens AG, Erlangen, Germany). To minimize head movements within the head coil, foam rubber was used to fixate the participant’s head. A T1-weighted structural scan of the whole brain was obtained using the sequence MPRAGE (matrix size = 256 × 256, 160 partitions, 1 mm^3^ isotropic voxels, TA = 5:17 m, TR = 2300 ms, TE = 3.93 ms, flip angle = 8°, FOVRO = 256, FOVPE = 224, PAT = 2, PAT mode = GRAPPA) that served as the anatomical reference for MEG source localization.

### Whole-Head Magnetoencephalography (MEG)

Neuromagnetic activity was recorded while participants were seated in upright position using a 275-sensor whole-head MEG system (CTF MEG® by MISL, Coquitlam, BC, Canada) recording at a sampling frequency of 3906.25 Hz and bandwidth of 0–976Hz. Third order synthetic gradiometer configuration was used to attenuate environmental noise. Before MEG recordings, three fiducial localization coils were placed at the nasion, left and right pre-auricular area to determine the participant’s head position during the MEG recording. Coil positions were continuously measured and locations stored to allow for offline co-registration with T1-weighted MR images. During MEG recordings, participants were asked to minimize head and body movements to avoid movement artifacts.

### Transcranial Alternating Current Stimulation in the MEG Environment

tACS was delivered using a commercial transcranial brain stimulator (DC-STIMULATOR MR^®^, NeuroConn GmbH, Ilmenau, Germany). Two radio-translucent stimulation electrodes (5 cm × 7 cm) were placed according to the international 10–20 system over electrode positions Fpz overlying the medial frontal lope and Pz overlying the parietal cortex (Thompson and Thompson, 2003). Impedance between the stimulation electrodes was maintained below 20 kΩ using a conductive paste (Ten20^®^, D.O. Weaver, Aurora, CO, USA). The battery driven stimulator device was located outside the magnetically shielded room and the electric currents were delivered via a twisted pair of wires. tACS was applied at a peak-to-peak current intensity of 2 mA oscillating at 220 Hz (as in Witkowski et al., 2015), while the stimulation signal was modulated at each individual’s peak theta frequency previously assessed during the familiarization session. The stimulation signal was generated using Matlab and streamed to the DC-STIMULTOR MR^®^’s remote input channel. The stimulator device linearly converted the input voltage into a corresponding stimulator output current (1 V = 2 mA).

To avoid possible discomfort during the onset of tACS, the stimulation current was gradually ramped up from 0 to 2.0 mA over a period of 10 s before onset of block 2, and then kept constant for the whole time of block 2. At the end of block 2, tACS was ramped down from 2.0 to 0 mA over 10 s. For sham stimulation, no current was delivered throughout the session. The stimulator output signal was recorded along with the neuromagnetic activity using the MEG signal acquisition system so that phase-locking of reconstructed brain oscillations and the stimulator output signal could be calculated at a later stage. For sham stimulation, the stimulator output signal was recorded, but not converted into an actual output current. At the end of the session, each participant was asked to indicate whether tACS or sham stimulation was applied. To determine whether participants were able to distinguish their group assignment, a chi-square test was performed (see “Supplementary Material”).

### Whole-Brain Reconstruction of Source Activity

Recorded MEG data was low-pass filtered at 110 Hz using a 6th-order Butterworth filter and decimated by a factor of five. Synthetic Aperture Magnetometry (SAM) beamforming was used for reconstruction of source activity (SA) (Robinson and Vrba, 1999). The covariance matrix for all following processing steps was estimated based on the entire time-series. The weights for each voxel were estimated using the covariance matrix. Source signals were reconstructed by multiplying the estimated weights and preprocessed MEG data. SA was estimated at 5 mm resolution on a regular 3D grid using a single shell head model (Nolte, 2003).

### Identification of Each Individual’s Frontal Midline Theta (FMT) Peak Frequency

For identification of each individuals FMT peak frequency, MEG data recorded during the familiarization session was used to estimate each individual’s theta activity by reconstructing source signals previously band-pass filtered from 4 to 8 Hz using a 3rd order Butterworth filter. The voxel with the most significant 2-back task-related theta activity increase during the familiarization session was identified and the corresponding source signal’s frequency spectrum then estimated using fast Fourier transform (FFT). The frequency showing the maximum difference in signal power during the 2-back task in comparison to task-free intervals was then determined and used to set the modulation frequency of tACS in block 2.

### Assessment and Mapping of tACS Phase-Locked Frontal Midline Theta Activity (FMTΔPLV)

To detect tACS phase-locked activity, phase locking value (PLV) between the theta-modulated tACS signal envelope and oscillatory SA across the whole brain was computed. In preparation for this, the instantaneous phases of the tACS signal envelope and reconstructed SA of each voxel was estimated by applying a Hilbert transformation on source signals filtered from 4 to 8 Hz (6th order Butterworth filter). PLV was then computed as a function of instantaneous phase difference between the tACS signal envelope and oscillatory SA using the following equation:

(1) $$PLV=\frac{1}{N}\left|\sum_{n=1}^{N}e^{i(\Phi_{SA}\left(n\right)-\Phi_{SE}(n))}\right|$$

Where N is the number of sampled time points, Φ_SE_ and Φ_SA_ are the instantaneous phases at time point *n* of the stimulator’s output signal *(SE)* and the reconstructed SA respectively. PLV can range between 0 and 1, where a value close to 0 indicates random phase relationship while a value close to 1 indicates a fixed signal phase relationship.

Differences in PLV were calculated between block 1 and 2 (ΔPLV). Mapping of tACS phase-locked brain activity was performed after spatial blurring using a Gaussian kernel with 5 mm full width at half maxima. Voxels were identified that exhibited increased ΔPLV between group A and B using an independent sample Wilcoxon rank sum test. Significance level was set at *p* < 0.05 and results corrected for multiple comparisons. Clusters with volumes less than 1 mL were excluded. For visualization, topographic images of frontal, sagittal and transversal view points were generated and voxels color-coded depending on ΔPLV magnitude using AFNI (Cox, 1996).

To test whether tACS was associated with an increase of theta PLV, an independent sample Wilcoxon rank sum test was performed comparing ΔPLV of the voxel with the highest ΔPLV increase in group B with ΔPLV of this voxel in group A. Additionally, ΔPLV was calculated and plotted across different frequencies to assess frequency-specificity of tACS entrainment (see “Supplementary Materials”).

### Assessment and Mapping of Frontal Midline Theta Power (FMTΔPower)

MEG source activity was filtered from 4 to 8 Hz (6th order Butterworth filter) and Hilbert transformed. Task-related differences in FMT power were calculated as difference in power between rest state and 2-back task and contrasted between block 1 and 2 (FMTΔpower). For visualization, topographic images of frontal, sagittal and transversal viewpoints were generated and voxels were color-coded depending on their FMTΔPower using AFNI.

To investigate whether tACS resulted in lack of FMTΔPower increase, FMTΔPower of the same voxel selected for comparison of ΔPLV between groups was calculated. Values were compared between group A and B using an independent sample Wilcoxon rank sum test. Significance level was set at *p* < 0.05 and results corrected for multiple comparisons.

### Working Memory Performance

Response accuracy (nAcc) per block was calculated as percentage of correct responses normalized by the group median. To rule out difference in WM performance between groups in block 1, an independent sample Wilcoxon rank sum test with “response accuracy” was performed. As a second step, differences in WM performance between block 1 and block 2 (ΔnAcc) were compared between group A and B using an independent sample Wilcoxon rank sum tests. Significance level was set to *p* < 0.05.

## Results

### Source Localization of tACS Phase-Locked Frontal Midline Theta Activity (FMTΔPLV)

Localization of tACS phase-locked FMT oscillations as measured by ΔPLV in block 2 identified the right and left medial and superior frontal gyrus and ACC as cortical areas with the greatest impact of frontal theta tACS on FMT phase. These areas matched brain regions previously found to be involved in WM task execution showing WM demand-related FMTΔpower (Brookes et al., 2011, Figure 2). The voxel with the highest ΔPLV was located in the left PFC (Talairach coordinate [60, −6, 12]). ΔPLV at this voxel was significantly different between groups (group A: *Mdn* = 0.039; group B: *Mdn* = −0.001) evidencing that tACS, but not sham stimulation, resulted phase locking of FMT oscillations (Figure 3).

**Figure 2:**
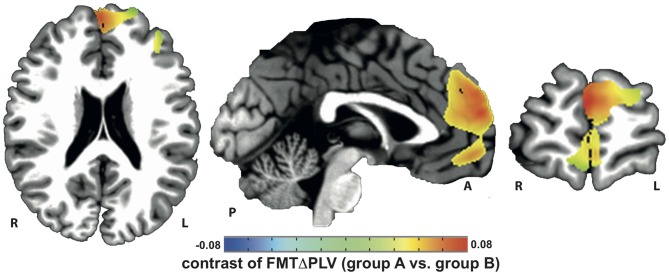
**Increase in tACS-dependent phase-locked brain activity was identified in frontal brain areas that included the prefrontal and anterior cingulate cortex (PFC/ACC) previously shown to be important for working memory (WM) task execution**.

**Figure 3:**
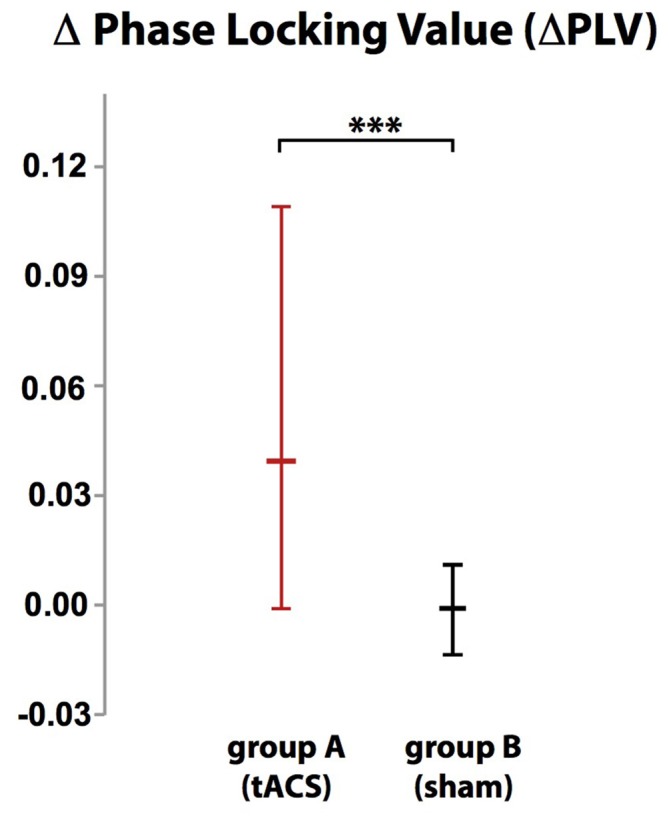
**While there was no difference in phase-locking value (ΔPLV) between blocks in group B (sham stimulation), ΔPLV increased in group A (tACS) documenting tACS-dependent manipulation of frontal midline theta (FMT) phase.** Error bars indicate 95% confidence intervals. ***p < 0.001.

### Assessment and Mapping of Frontal Midline Theta Power (FMTΔPower)

**Figure 4:**
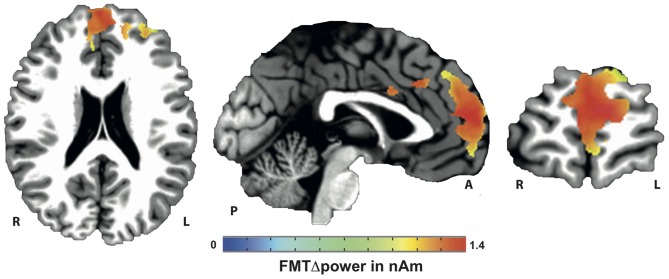
**Mapping of FMT power increase (FMTΔpower) during execution of a 2-back task**.

**Figure 5:**
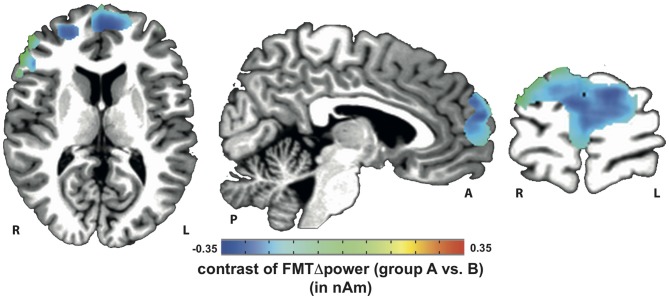
**Topographic map of voxels showing significant contrast in FMTΔpower when comparing 2-back task-related theta power increase during tACS vs. sham stimulation.** Lack of theta power increase was found throughout the whole *n*-back task-related frontal cortical areas.

**Figure 6:**
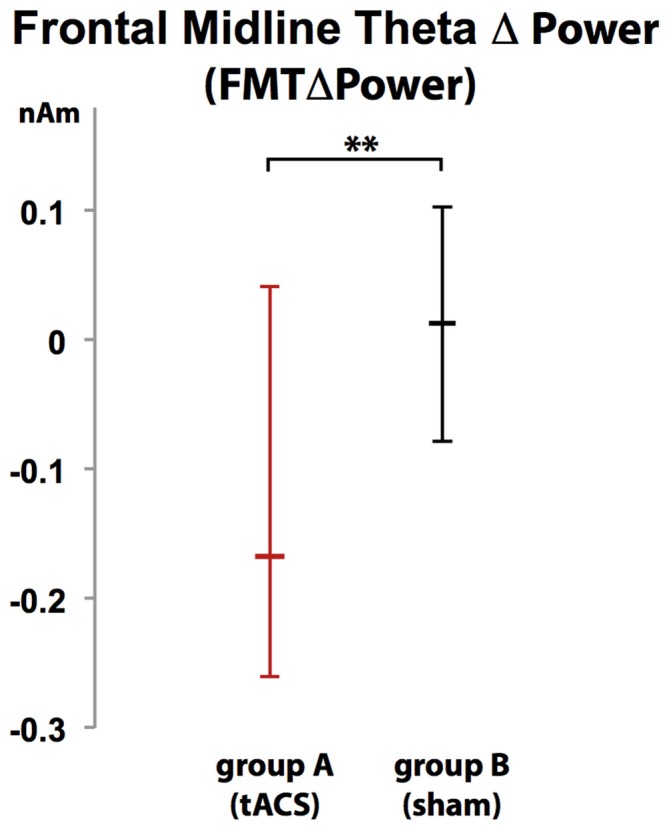
**Increase in FMT power (FMTΔpower) during execution of a 2-back task was significantly different in group A (tACS) compared to B indicating that frontal theta tACS results in lack of FMTΔpower shown to correlate with WM demand and performance.** Error bars indicate 95% confidence intervals. **p < 0.01.

Voxels showing WM task-related theta power increase were located in the left and right anterior cingulate gyrus, medial and superior frontal gyrus as well as left supplementary motor cortex (SMA; Figure 4). A topographic map based on the difference in FMTΔPower between group A and B showed that tACS-associated lack of power increase was widespread matching cortical areas previously identified to be active during execution of a 2-back task (Figure 5). An independent sample Wilcoxon rank sum test revealed a significant difference in FMTΔPower in the PFC (Talairach coordinate [60, −6, 12]) of group A (tACS; *Mdn* = −0.165) compared to group B (sham stimulation; *Mdn* = 0.014; *U*_(18)_ = 2.835, *p* = 0.005; Figure 6) indicating that frontal theta tACS influenced regulation of FMT power during execution of a 2-back task.

### Change in Working Memory Performance (ΔnAcc)

**Figure 7:**
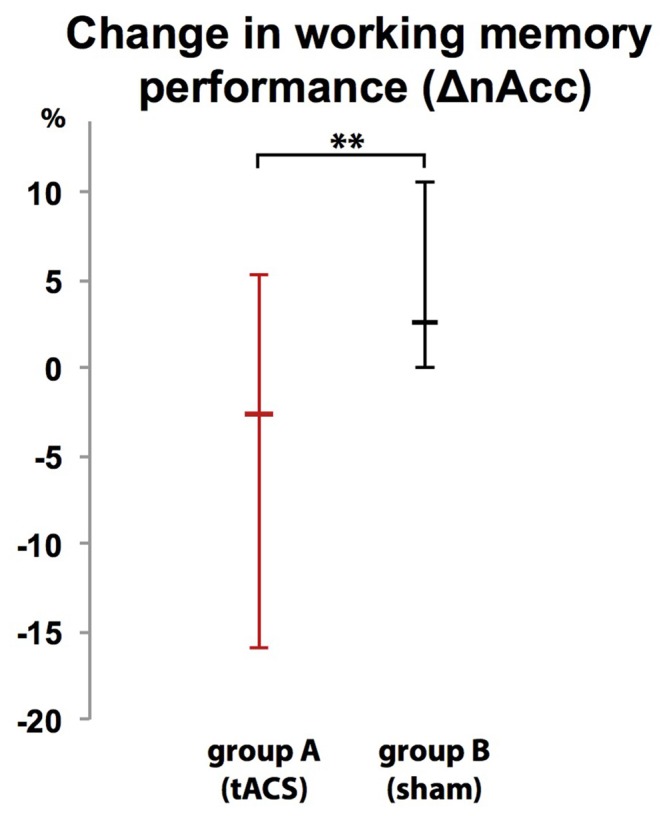
**Group-dependent difference in WM performance as measured as difference in correct responses in a 2-back task between a baseline block (block 1) and interventional block (block 2).** WM performance dropped when tACS was applied (group A) but not during sham stimulation (group B). Error bars indicate 95% confidence intervals. **p < 0.01.

Participants were unable to differentiate to which group (A or B) they were assigned (*χ*^2^ (2, *N* = 20) = 2.300, *p* = 0.986). While there was no difference in WM performance in block 1 (baseline block) between group A (tACS) and group B (sham stimulation; *U* = 1.35 *p* = 0.179, group A: *Mdn* = 95%, group B: *Mdn* = 95%), comparison of WM performance in block 2 between groups showed that tACS but not sham stimulation decreased response accuracy (ΔnAcc; sham; *U*_(18)_ = 2.665, *p* = 0.008, group A: *Mdn* = 2.63%, group B: *Mdn* = −2.63%; Figure 7).

## Discussion

This is the first study that assessed WM task-related FMT oscillations using whole-head MEG during simultaneous application of frequency-tuned tACS. Besides showing that FMT phase can be purposefully manipulated using frontal lobe tACS delivered at individual theta frequency, this study provides evidence that interference with endogenous FMT phase regulation, shown to be important for maintenance of temporal order information, is associated with lack of FMTΔpower and interferes with normal WM performance in an n-back task. While there was no difference in WM task-related FMTΔpower and WM performance (ΔnAcc) between both groups in block 1 in absence of any stimulation, application of frequency-tuned tACS but not sham stimulation reduced WM task-related FMTΔpower and lowered accuracy of WM performance in block 2. This finding suggests that interference with endogenous FMT phase regulation undermines stabilization and maintenance of temporal order information required for correct responses in an n-back task. Consequently, as inhibition of competing representations is not required, task-related FMTΔpower during task execution is reduced, as shown for block 2 of group A (tACS) in our study. While our findings underline the importance of endogenous phase regulation for WM performance in an n-back task, interference with phase-regulation during other WM tasks in which maintenance of temporal order information is not required might not influence task performance or affect signal power modulation of brain oscillations, an issue that should be investigated in future studies. It is conceivable that interference with endogenous phase regulation that disrupts dysfunctionally coupled activity between relevant brain areas might, on the contrary, improve WM-related brain function, e.g., in the context of brain disorders such as depression, epilepsy, Alzheimer’s or Parkinson’s disease (Voytek and Knight, 2015). Better characterizations of these dysfunctional neural circuits and brain areas that can be targeted by transcranial brain stimulation to modulate these circuits are needed, however.

While in this study tACS was applied as open-loop stimulation, i.e., phase of the tACS signal was not adjusted to and independent of the phase of the endogenous brain oscillations, closed-loop stimulation in which brain stimulation is adjusted to the physiological parameters in real-time may allow for more specific and versatile modulation of brain function. Correctly adjusted, closed-loop tACS may not disrupt maintenance of temporal order information, but, on the contrary, stabilize such information, which should be reflected in an increase of task-related FMT power and improved n-back task performance. Such closed-loop paradigm allowing for precise tuning of tACS extending the currently available non-invasive tools to target brain oscillations would not only open new avenues to investigate the link between brain physiology and human behavior, but might also improve treatment options of brain disorders in which coordination of activity between different brain regions is disturbed.

Mapping of tACS phase-locked (entrained) FMT oscillations showed that tACS phase locking occurred mainly in areas previously identified as generators of FMT, particularly the medial PFC and ACC. The individual role of the PFC and ACC in modulating FMT phase and power is still widely unknown. While attention coordination and error monitoring was attributed to ACC activation (Gehring and Knight, 2000), sustaining attention on task goals (MacDonald et al., 2000) and inhibiting competing cortical representations (Norman et al., 2007) was predominantly attributed to PFC activation (Munakata et al., 2011). It is not fully understood, though, how such activation (often measured as blood-oxygen-level dependent (BOLD) contrast using functional magnet resonance imaging) relates to modulations of theta activity. While our data shows that tACS-based PLV-increase affects both ACC and PFC, lack of FMT power increase during the 2-back task was only found in PFC, but not ACC. This indicates a differential role of how PFC and ACC each modulate FMT, and we’d speculate that FMT phase is influenced by both, while FMT power is predominantly modulated by PFC.

As the phase information of the tACS signal was sustained and spread across the whole task-related network independent of the tACS current flow (passing mainly through superficial cortical layers towards the parietal electrode, Laakso and Hirata, 2013), further evaluation of this phenomenon might be of great importance for the development of new transcranial stimulation strategies specifically targeting brain oscillations generated in subcortical areas. The property that tACS targeting a node of a widespread functional network can influence oscillatory activity in areas distant from the stimulation electrode was recently also shown for tDCS (Garcia-Cossio et al., 2015). Interestingly, tACS phase locking was predominantly restricted to areas generating endogenous brain oscillations at frequencies similar to the tACS signal (frontal theta oscillations in our study). The fact that cortical areas underneath the parietal reference electrode did not show such differences in theta PLV or other frequencies during tACS (see “Supplementary Data Analysis”) further corroborates this finding.

Whereas some previous brain stimulation studies indicated that entrainment at a specific target frequency can be associated with an increase in power at this very frequency, we did not find such entrainment-related power increase. This may relate to the different stimulation protocols that were used. Having shown that a FFT of reconstructed MEG source activity (SA) recorded during mono-sinusoidal (classical) tACS exhibits a large spectral power peak at the stimulation frequency, we concluded that noise cancelling features of SAM beamforming and possibly all linearly constrained minimum variance (LCMV) beamformers fail to reliably distinguish between physiological brain activity and stimulation-dependent artifacts of classical tACS (Witkowski et al., 2015). Additionally, Noury et al. (2016) have recently shown that heartbeat and respiration non-linearly modulate stimulation artifacts which may have resulted in misinterpretation of data acquired during classical tACS. We, thus used amplitude-modulated tACS in our study to overcome this problem. Future studies need to further elucidate the differences in the physiological effects of entrainment induced by mono-sinusoidal vs. amplitude-modulated tACS. The demonstrated tACS-dependent phase entrainment in the whole FMT network using amplitude-modulated tACS indicates, though, that increase in power related to mono-sinusoidal tACS, if not artifactual, may relate to other mechanisms than phase entrainment.

Whereas classical tACS, particularly when applied over frontal brain areas, can be associated with cutaneous retinal activation (Schutter, 2015), none of the study participants reported any photic stimulation or tACS-related discomfort leaving them unable to distinguish actual tACS from sham stimulation. Differences in specific physiological effects between classical mono-sinusoidal and amplitude-modulated tACS should be studied in future investigations. While not addressed in our study, recent studies indicate that gender can influence NIBS effects on WM performance (Meiron and Lavidor, 2013), an important issue to be considered in future studies.

After rhythmic transcranial current stimulation was already successfully used to modulate function of the motor domain, e.g., to suppress Parkinsonian resting tremor (Brittain et al., 2013), our study suggests that tACS might be a powerful tool to targeted cognitive control and adaptive behavior.

## Author Contributions

SRS, BSC, MW, CB designed the study. BSC, MW, SRS collected data. SRS, BSC, MW, CB analyzed data. SRS, BSC, MW, CB, SER, JB, LGC and NB interpreted data and did the literature search. SRS, BSC, MW wrote the manuscript and created the figures. SRS, BSC, MW, CB, SER, JB, LGC and NB edited the manuscript. Competing interests: All authors declare no competing financial interests. Data and materials availability: all raw data can be made available upon request.

## Conflict of Interest Statement

The authors declare that the research was conducted in the absence of any commercial or financial relationships that could be construed as a potential conflict of interest.
