# Supplementary material for: tACS Phase Locking of Frontal Midline Theta Oscillations Disrupts Working Memory Performance
Source: Front Cell Neurosci. 2016 May 6;10:120. doi: 10.3389/fncel.2016.00120 (PMC4858529; doi:10.3389/fncel.2016.00120)
Supplement: Supplementary file 1 [file DataSheet_1.pdf]

Supplementary material

Supplementary data analysis

Placebo-control of stimulation

To rule out that participants could distinguish between active tACS and sham stimulation, all subjects were asked at the end of each session whether they believed to have received active brain stimulation throughout the session (*active*) or not (*inactive*) (Supplementary Table S2). A chi-square test was performed to determine whether the groups were able to distinguish active and inactive stimulation across sessions. The ability to distinguish was equally distributed in the population, $\chi^2_{(2,\ N\ =\ 20)} = 2.300$, $p = .986$ suggesting that participants were unable to reliable distinguish whether active tACS was applied or not.

Modulation of PLV underneath the parietal electrode in other frequency bands

To investigate whether theta tACS applied over the parietal lobe results in PLV increase in other frequency bands, ΔPLV was estimated at a frequency resolution of 0.5 Hz in each band (delta: 0.1-4Hz; alpha: 9-15Hz; and beta: 15-30Hz) and compared between groups using a two-sided Student's t-test. Resulting p-values were corrected for multiple comparisons using Bonferroni correction. Theta tACS did not result in any significant PLV increase in any of the tested frequency bands (delta: $p = .4703$; alpha: $p = .4264$; beta: $p = .1866$).

Supplementary Tables

Table S1: Theta peak frequencies across participants

**Participant (group A)**	**Theta peak frequency**
1	6.6 Hz
2	5.9 Hz
3	6.2 Hz
4	5.8 Hz
5	6.6 Hz
6	5.4 Hz
7	5.5 Hz
8	6.5 Hz
9	5.2 Hz
10	6.5 Hz
**Participant (group B)**	**Theta peak frequency**
1	6.2 Hz
2	5.2 Hz
3	5.9 Hz

4	4.9 Hz
5	4.9 Hz
6	5.6 Hz
7	6.4 Hz
8	6.3 Hz
9	6.9 Hz
10	5.3 Hz

Table S2: Response of participants when asked whether they believed to have received active or inactive brain stimulation during block 2

**Participant (group A)**	**Response** (active / inactive)
1	Inactive
2	Active
3	Inactive
4	Inactive
5	Active
6	Inactive
7	Inactive
8	Inactive
9	Active
10	Inactive
**Participant (group B)**	**Response** (active / inactive)
1	Inactive
2	Inactive
3	Inactive
4	Active
5	Inactive
6	Active
7	Active
8	Active
9	Inactive
10	Inactive

Supplementary Table S1: Placebo-control of stimulation type. Participants were asked at the end of each session whether they believed to have received active stimulation during block 2 (*active*) or not (*inactive*). Responses were not different between stimulation types suggesting that participants were unable to reliable distinguish whether active stimulation was applied or not.

Supplementary Figures

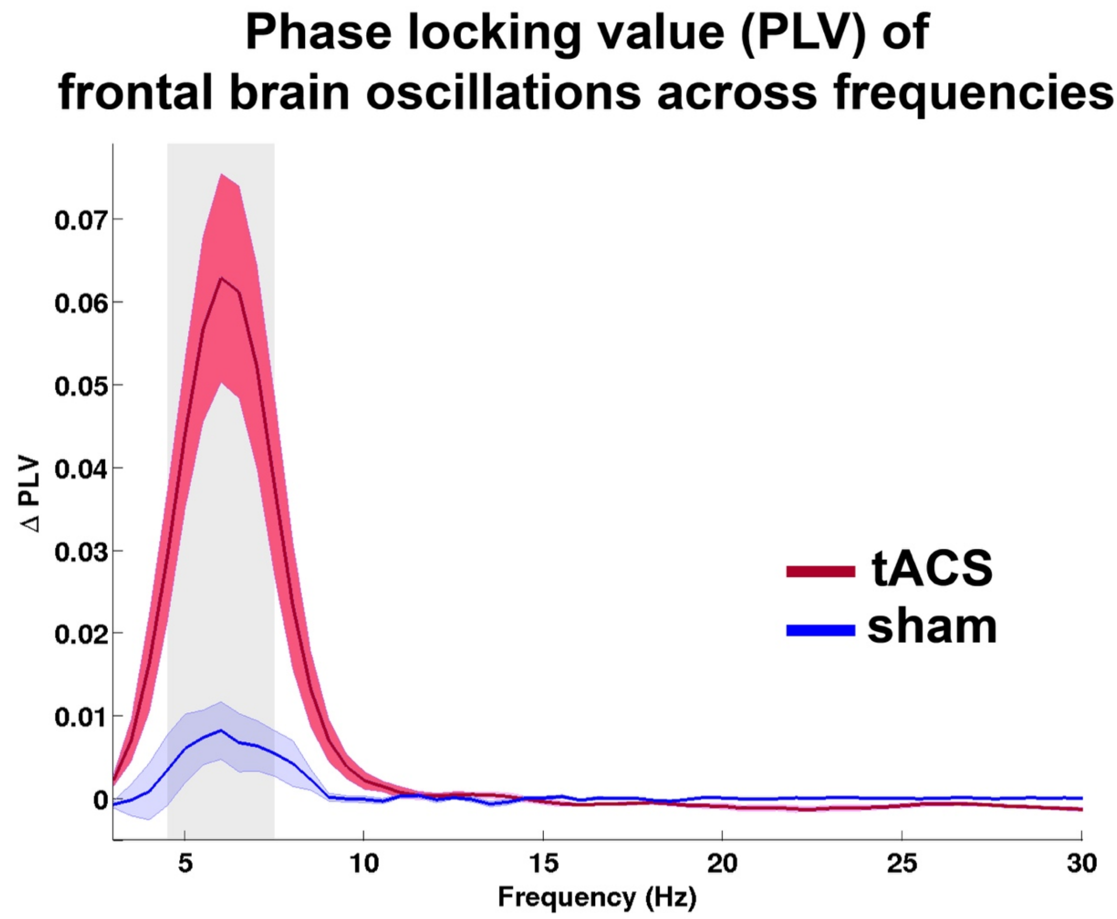

Fig. S1: Frequency specificity of transcranial alternating current stimulation (tACS)-dependent phase locking of brain oscillations. Difference in phase locking value (ΔPLV) between block 1 (baseline block) and block 2 (intervention block) in group A (tACS, red line) and group B (sham, blue line) across different frequency bands in the left prefrontal cortex (PFC) (Talairach coordinate [60 -6 12]). Shaded areas indicate standard error (SE). ΔPLV increased only in theta band and matched with the frequency of the amplitude modulated tACS signal's envelope oscillating at each individual's frontal midline theta (FMT) frequency. The grey shaded area indicates a significance level of $p < .01$.
